# Optimizing patient outcome in intracranial tumor surgery: a detailed prospective study of adverse events and mortality reduction strategies in neurosurgery

**DOI:** 10.1007/s00701-024-06008-y

**Published:** 2024-03-08

**Authors:** Pavlina Lenga, Helena Kleineidam, Andreas Unterberg, Philip Dao Trong

**Affiliations:** 1 Department of Neurosurgery, Heidelberg University Hospital, Im Neuenheimer Feld 400, 69120 Heidelberg, Germany; 2 Medical Faculty of Heidelberg University, Heidelberg, Germany

**Keywords:** Adverse events, Tumor surgery, Morbidity, Intracranial tumors

## Abstract

**Introduction:**

Brain tumor surgery represents a critical and high-risk area within the field of neurosurgery. Our study aims to offer a comprehensive analysis of adverse events (AEs) from a prospectively maintained database at a leading neurosurgical tertiary center, with a specific focus on different types of tumor entities.

**Methods:**

From January 2022 to September 2023, our study focused on adult patients, who underwent surgery for intracranial tumors. Each patient in this demographic was thoroughly assessed for adverse events (AEs) by their attending physicians at discharge. An AE was defined as any event occurring within the first 30 days post-surgery.

**Results:**

A total of 1173 patients with an average age of 57.4 ± 15.3 years underwent surgical procedures. The majority of these surgeries were elective, accounting for 93.4% (1095 out of 1173), while emergency surgeries constituted 13.9% (163 out of 1173). The incidence of surgery-related AEs was relatively low at 12.7%. The most common surgical indications were meningioma and glioma pathologies, representing 31.1% and 28.2% of cases, respectively. Dural leaks occurred in 1.5% of the cases. Postoperative hemorrhage was a significant complication, especially among glioma patients, with ten experiencing postoperative hemorrhage and eight requiring revision surgery. The overall mortality rate stood at 0.8%, corresponding to five patient deaths. Causes of death included massive postoperative bleeding in one patient, pulmonary embolism in two patients, and tumor progression in two others.

**Conclusions:**

Surgical interventions for intracranial neoplasms are inherently associated with a significant risk of adverse events. However, our study’s findings reveal a notably low mortality rate within our patient cohort. This suggests that thorough documentation of AEs, coupled with proactive intervention strategies in neurosurgical practices, can substantially enhance patient outcomes.

## Introduction

Brain tumor surgery has always been considered a critical and high-risk domain within neurosurgery. In the last decades, significant advancements have been made, including the development of new imaging techniques for mapping critical brain fibers, implementation of intraoperative navigation systems, and neuromonitoring [16, 17]. Despite these advancements, adverse events (AEs) remain an inherent risk. Effective documentation of AEs is essential to maintain and enhance the quality of healthcare delivery. Consequently, morbidity and mortality conferences (MMCs) have become increasingly important for reviewing and preventing recurrent AEs [19]. Several classification systems have been established with the common goal of assessing the occurrence and severity of AEs [7, 20, 32]. This is particularly crucial in brain tumor surgery, where surgical AEs can lead to permanent disability, extended hospital stays, or unplanned readmissions—all contributing to increased healthcare costs [6, 16, 36]. The incidence of AEs in neurosurgical care is emerging as a primary indicator for quality assessment. This trend underscores the need for transparent informed consent processes for patients and serves as a critical marker for modern hospital management to monitor and mitigate risks [10, 25]. Currently, our understanding of adverse events (AEs) following brain tumor surgery is primarily derived from administrative or retrospective data sources, which do not fully capture the real-world scenario [3, 9, 21, 29, 30]. Our study group’s prior research suggests that adequately documenting AEs in a prospective setting may reduce their incidence [2, 22], especially with the deployment of established scores such as the Clavien-Dindo classification (CDC). The implementation of these systems in brain tumor surgery could be a crucial tool for tailoring risk management strategies to align with the specific characteristics of each patient and the type of tumor involved.

To address this data deficiency, our study was designed to provide an extensive analysis of a prospectively collected database from a major neurological tertiary center. We specifically focus on brain tumor surgery, aiming to refine current quality metrics and improve patient outcomes in relation to different tumor types.

## Methods

This study was conducted as a prospective investigation at a distinguished tertiary care hospital spanning between January 2022 and September 2023. The research was approved by the local ethics committee under reference number S-425/2022, adhering to the Declaration of Helsinki’s guidelines. As reported by Dao Trong et al. and Lenga et al. [2, 22], our research team included 15 board-certified neurosurgeons and 18 neurosurgical residents who were responsible for the meticulous recording and updating of patient data in our dedicated database.

Upon discharge, patients were given a form to report any postoperative adverse events (POPAE), which was filled out by the attending physician. These forms underwent a thorough review by a senior supervising neurosurgeon before the data were entered into our database. In instances of patient readmission within 30 days following the initial surgery, the treatment team received an automatic notification. All complex cases were subjected to comprehensive discussions during multidisciplinary morbidity and mortality conferences (MMC) involving the entire neurosurgical staff.

For the purpose of this analysis, we extracted and examined consecutive data specifically from adult patients with intracranial tumors, while pediatric cases were not included in the study.

Adverse events (AEs) were classified into several categories: wound events, postoperative infections, cerebrospinal fluid (CSF) fistulas, new neurological deficits, postoperative hemorrhage, and failure to meet surgical objectives. We defined elective surgery as any procedure scheduled at least 1 day in advance. In contrast, non-elective surgery encompassed emergency procedures and surgeries that required revision.

### Definitions

- Wound event: This term encompasses any superficial or deep wound healing complications, inclusive of those with concurrent infection.
- Postoperative infections: These are specifically identified as occurrences of meningitis, abscess formation, or empyema post-surgery.
- CSF fistula: Defined as any instance of internal or external cerebrospinal fluid leakage, including rhinoliquorrhea.
- Implant malfunction/CSF shunt dysfunction: Includes any form of valvular dysfunction, mechanical obstruction, or catheter occlusion.
- Malpositioning of implanted material: This pertains to the incorrect placement of ventricular or abdominal CSF catheters, pedicle screws, rods, or intervertebral cages.
- New neurological deficit: Described as any neurological impairment emerging postoperatively that was not evident prior to surgery, or any exacerbation of existing deficits.
- Rebleeding: Defined as bleeding into the resection cavity, subdural space, or soft tissue that precipitates a new neurological deficit or necessitates further surgical intervention.
- Surgical goal not achieved: Refers to instances where the predefined objectives of the surgery were not fully accomplished, including incomplete resections.
- Mortality was defined as any cause of death within 30 days post-surgery.

The Clavien-Dindo classification was deployed to provide the gravity of single adverse events [7].

### Statistics

Quantitative categories were articulated in numerical counts and corresponding percentages. Continuous datasets, authenticated for normal distribution via the Shapiro–Wilk test, were expressed in means ± standard deviations. All analytical endeavors were executed utilizing SPSS version 24.0.0.0 (IBM Corp., Armonk, NY, USA).

## Results

### Study population and baseline characteristics

**Fig. 1:**
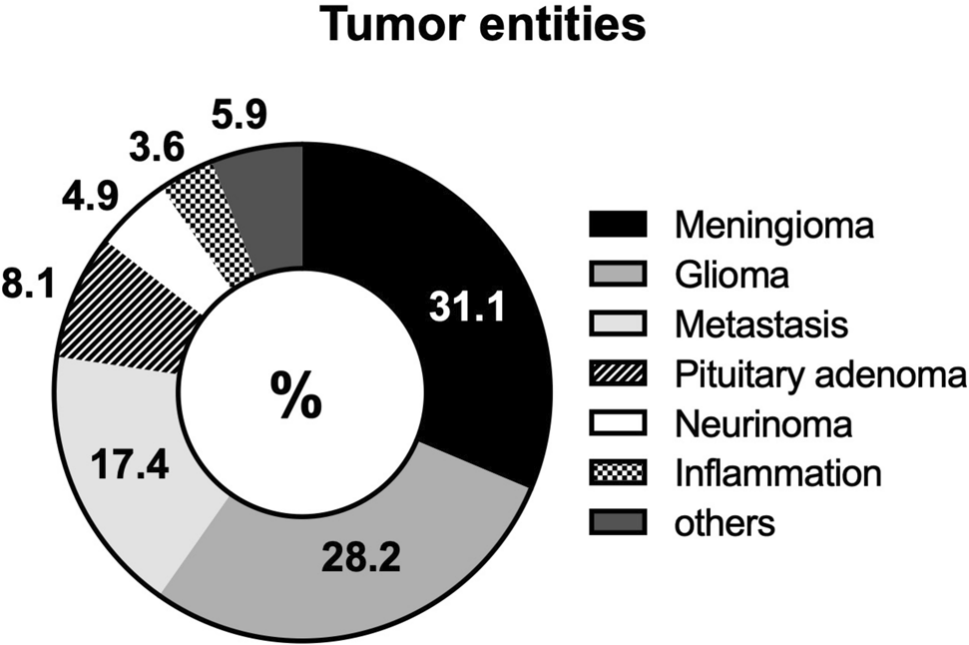
Overview of intracranial tumor entities of study population

**Fig. 2:**
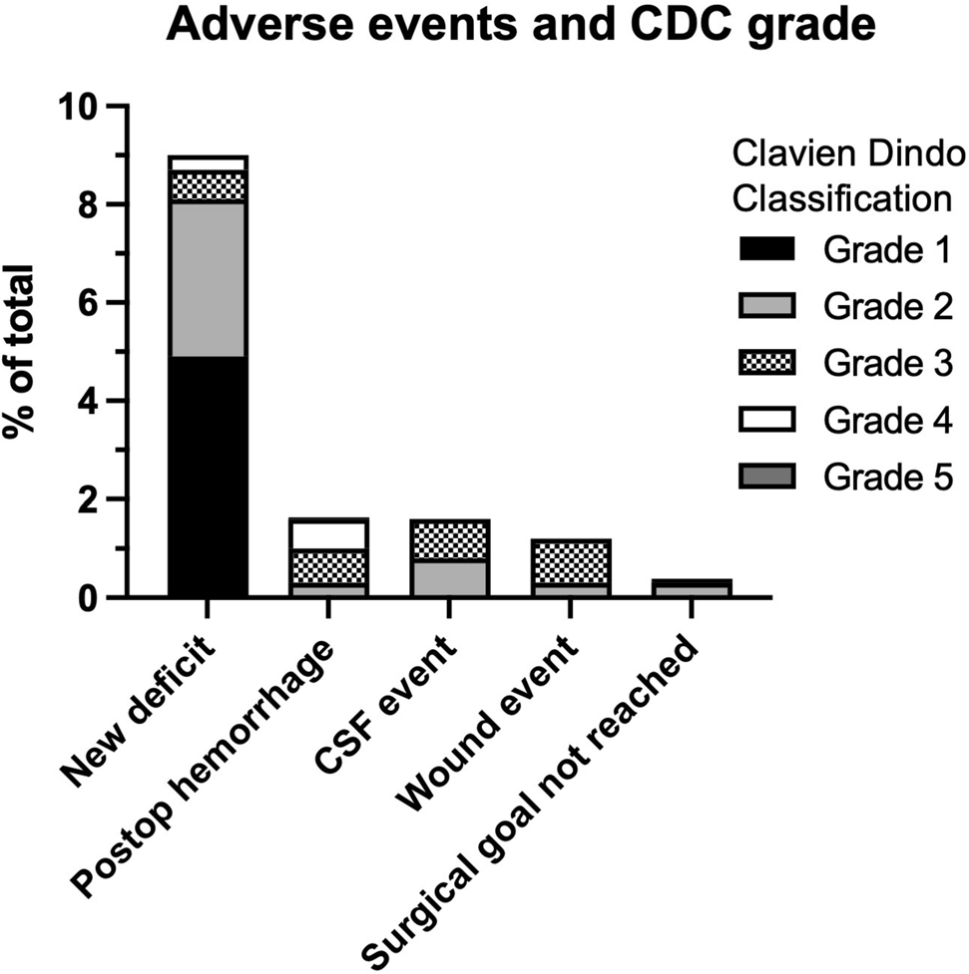
Classification of adverse events according to the Clavien-Dindo system

A total of 1173 patients with a mean age of 57.4 ± 15.3 years (range: 18–93 years) underwent surgery between January 2022 and September 2023. Elective surgery was performed in 93.4% (1095/1173) of patients, while emergency surgery was performed in 13.9% (163/1173). The overall rate of surgery-related AEs was relatively low (12.7%). The overall revision rate was 4.2%. Meningioma and glioma pathologies were the most common reason for surgery (31.1; 28.2%, respectively). A detailed description of the study population is presented in Table 1 and Fig. 1. The occurrence of AEs was categorized by employing the CDC system as displayed in Fig. 2.

**Table 1:** Study population baseline characteristics

	*n* = 1173	%
Age, years (mean, SD)	57.4 (15.3)	–––
Sex (*n*, %)		
Male	500	42.6
Female	673	57.4
Non-elective	78	6.6
Elective	1095	93.4
Supratentorial	515	43.9
Infratentorial	658	56.1
Pathology		
Meningioma	365	31.1
Glioma	331	28.2
Metastasis	204	17.4
Pituitary adenoma	95	8.1
Neurinoma	58	4.9
Inflammation	42	3.6
Hemangioblastoma	15	1.3
Ependymoma	11	0.9
Sarcoma	10	0.9
Chordoma	1	0.9
Epidermoid tumor	9	0.8
Embryonal tumor	7	0.6
Pineal region tumors	6	0.5

*SD*, standard deviation

### Occurrence of surgery-related AEs

#### Wound events

In our study, wound events were observed in a diverse group of patients with different types of intracranial tumors. Specifically, such events occurred in seven (2.1%) patients diagnosed with glioma. Additionally, three patients each with meningioma and metastasis respectively also experienced wound events. Further, one patient with ependymoma and another with hemangioblastoma were observed to have similar complications. Of these cases, revision surgery was necessary in six of the seven glioma patients (1.8%). In contrast, all three (0.8%) meningioma patients, two (1.5%) patients with metastasis, and one (6.7%) patient with hemangioblastoma required surgical intervention for their wound events.

#### Dural leak

The overall prevalence of dural leaks in our study was found to be 1.5%. Specifically, cerebrospinal fluid (CSF) leakage occurred in seven (2.1%) patients with gliomas, out of which only two cases necessitated revision surgery. Additionally, four (6.9%) patients with intracranial neurinomas experienced CSF leakage, with half of these cases requiring surgical intervention.

#### Postoperative hemorrhage

Postoperative hemorrhage was a notable complication, particularly in patients with gliomas. Specifically, ten (3.0%) glioma patients experienced postoperative bleeding, and eight of these underwent revision surgery. In addition, among the patients with meningioma, four (1.1%) suffered from postoperative hemorrhage, with three requiring surgical revision. All three (5.2%) neurinoma patients who presented with post-surgical bleeding were also revised. Furthermore, two (1.1%) patients with metastases and one (7.1%) patient with an inflammatory condition required revision surgery due to postoperative hemorrhage.

#### New neurological deficit

Postoperatively, 3.2% of the cases developed new neurological deficits. Specifically, among 34 (10.3%) patients with gliomas, new postoperative neurological deficits were observed, and one of these patients required surgical revision. Similarly, 42 (11.5%) patients with meningiomas experienced postoperative deficits, with one undergoing surgical revision.

#### Surgical goal not achieved

Surgical goals were not met in a small number of cases: one patient with a meningioma (0.3%), one patient with a pituitary adenoma (10.5%), one patient with metastasis (1.0%), and one patient with an epidermoid tumor (11.1%). However, none of these patients required revision surgery due to this issue.

#### Secondary unplanned admissions to IMC or ICU and mortality rates

In 3.9% of the cases, a secondary admission to the Intensive Care Unit (ICU) or Intermediate Care (IMC) was necessary post-surgery due to the occurrence of AEs. The overall mortality rate was 0.8%, accounting for a total of five patient deaths. The causes of mortality included massive postoperative hemorrhage in one case, pulmonary embolism in two cases, and tumor progression in another two instances. A comprehensive description of all surgery-related adverse events (AEs) can be found in Table 2.

**Table 2:** Summary of surgery-related adverse events

	*n*	%	Revision surgery	%
Wound event				
Glioma	7	2.1	6	1.8
Meningioma	3	0.8	3	0.8
Ependymoma	1	9.1	0	0.0
Neurinoma	0	0.0	0	0.0
Pituitary adenoma	0	0.0	0	0.0
Pineal region tumors	0	0.0	0	0.0
Metastasis	3	1.5	2	1.0
Hemangioblastoma	1	6.7	1	6.7
Sarcoma	0	0.0	0	0.0
Chordoma	0	0.0	0	0.0
Epidermoid tumor	0	0.0	0	0.0
Embryonal tumor	0	0.0	0	0.0
Inflammation	0	0.0	0	0.0
Dural leak				
Glioma	7	2.1	2	0.6
Meningioma	2	0.5	2	0.5
Ependymoma	1	9.1	0	0.0
Neurinoma	4	6.9	2	3.4
Pituitary adenoma	1	10.5	0	0.0
Pineal region tumors	1	16.7	1	16.7
Metastasis	1	1.0	1	1.0
Hemangioblastoma	1	6.7	1	6.7
Sarcoma	0	0.0	0	0.0
Chordoma	0	0.0	0	0.0
Epidermoid tumor	0	0.0	0	0.0
Embryonal tumor	0	0.0	0	0.0
Inflammation	0	0.0	0	0.0
Postoperative hemorrhage				
Glioma	10	3.0	8	2.4
Meningioma	4	1.1	3	0.8
Ependymoma	0	0.0	0	0.0
Neurinoma	3	5.2	3	5.2
Pituitary adenoma	0	0.0	0	0.0
Pineal region tumors	0	0.0	0	0.0
Metastasis	2	1.1	2	1.1
Hemangioblastoma	0	0.0	0	0.0
Sarcoma	0	0.0	0	0.0
Chordoma	0	0.0	0	0.0
Epidermoid tumor	0	0.0	0	0.0
Embryonal tumor	0	0.0	0	0.0
Inflammation	1	7.1	1	7.1
Surgical goal not achieved				
Glioma	0	0.0	0	0.0
Meningioma	1	0.3	1	0.3
Ependymoma	0	0.0	0	0.0
Neurinoma	0	0.0	0	0.0
Pituitary adenoma	1	10.5	0	0.0
Pineal region tumors	0	0.0	0	0.0
Metastasis	1	1.0	0	0.0
Hemangioblastoma	0	0.0	0	0.0
Sarcoma	0	0.0	0	0.0
Chordoma	0	0.0	0	0.0
Epidermoid tumor	1	11.1	0	0.0
Embryonal tumor	0	0.0	0	0.0
Inflammation	0	0.0	0	0.0
New neurological deficit				
Glioma	34	10.3	1	0.3
Meningioma	42	11.5	3	0.8
Ependymoma	0	0.0	0	0.0
Neurinoma	9	15.5	2	3.4
Pituitary adenoma	3	3.2	0	0.0
Pineal region tumors	1	16.7	0	0.0
Metastasis	2	1.1	2	1.1
Hemangioblastoma	1	6.7	1	6.7
Sarcoma	0	0.0	0	0.0
Chordoma	0	0.0	0	0.0
Epidermoid tumor	0	0.0	0	0.0
Embryonal tumor	1	14.3	1	14.3
Inflammation	0	0.0	0	0.0
Secondary transfer to IMC or ICU				
Glioma	12	3.6	––	––
Meningioma	10	2.7	––	––
Ependymoma	0	0.0	––	––
Neurinoma	2	3.5	––	––
Pituitary adenoma	4	4.2		
Pineal region tumors	1	6.7	––	––
Metastasis	9	4.4	––	––
Hemangioblastoma	2	13.3	––	––
Sarcoma	1	10.0	––	––
Chordoma	0	0.0	––	––
Epidermoid tumor	1	11.1	––	––
Embryonal tumor	1	14.3	––	––
Inflammation	3	7.1	––	––
Death				
Supratentorial	3	0.6	–––-	–––-
Infratentorial	2	0.3	–––-	–––-

*IMC*, intermediate care unit; *ICU*, intensive care unit

#### Occurrence of surgery-associated medical complications

The overall incidence of surgery-associated medical complications was 7.0% (Table 3). In patients with supratentorial intracranial tumors, the most common events were pneumonia and pulmonary embolism, each occurring in 1.7% of cases. Similarly, in patients with infratentorial tumors, the most prevalent AEs were pulmonary embolism and delirium, also with an occurrence rate of 1.7% each.

**Table 3:** Summary of surgery-associated medical complications

	*n*	%
Pneumonia	19	23.2
COVID-19	8	9.8
Pulmonary embolism	20	24.4
Hypokalemia	6	7.3
Hyponatremia	7	8.5
Delir	14	17.1
Sepsis	2	2.4
Urinary tract infection	6	7.3

## Discussion

Surgical intervention for intracranial tumors is associated with a notable risk of AEs. The identification and management of these AEs are crucial for evaluating the efficacy of quality measures in neurosurgery. In this context, there is a concerted effort to enhance safety protocols to diminish the rates of mortality and morbidity associated with these surgical procedures. To our knowledge, our study is pioneering in its detailed examination of AEs following brain tumor surgery, specifically in relation to various types of intracranial pathologies. This analysis is grounded in a comprehensive and prospectively compiled database. Our findings indicate a relatively low incidence of AEs at 12.7%, with the need for revision surgeries in 4.2% of the cases. The most common AEs observed were dural leaks and the emergence of new neurological deficits, consistently noted across different groups. Mortality rates were found to be minimal at 0.8%, with causes including massive postoperative bleeding in one instance, pulmonary embolism in two cases, and tumor progression in another two instances. Additionally, the incidence of AEs not directly related to the surgery was found to be comparatively low at 7.0%, suggesting effective and meticulous postoperative care.

In their retrospective single-center study, Schipmann et al. reported a 24.5% rate of surgery-related AEs in a cohort of 2511 patients with brain tumor pathologies. The most common complication was CSF leakage, with surgical site infections and postoperative hematomas also being notable. Glioma was identified as the most frequent tumor type, with an incidence of 42.0% [29]. These findings are in line with Meyer et al.’s report of a 26.1% occurrence of AEs, with a significant proportion (8.4%) being severe or potentially life-threatening [25]. Similarly, a retrospective study by Lonjaret et al. on 167 brain surgery patients reported a 16.0% prevalence of AEs, predominantly in cases of malignant gliomas [24]. Contrasting with these findings, our study reveals a notably lower overall AE rate of 12.7%. This rate is particularly striking given the similar composition of our study cohort, where gliomas and meningiomas were the most frequent intracranial neoplasms. The observed diminution in the incidence of surgically induced AEs in our analysis may be attributable to the methodological rigor employed in the construction of our database. This database is meticulously configured to facilitate granular segregation of surgical AEs, thereby enabling the extrapolation of authentic prevalence metrics in real-world scenarios. Consequently, our dataset ostensibly provides a more veracious and comprehensive reflection of outcomes in comparison to antecedent research endeavors. Moreover, the meticulous process of AE documentation in our study, involving thorough recording by treating physicians and subsequent review by two attending doctors, might have minimized the potential for human error, thereby ensuring a more precise account of AEs.

Gliomas emerged as a predominant intracranial pathology in our study, with an overall surgery-related adverse event (AE) prevalence of 12.7%. Notably, a smaller proportion of these cases, 4.5%, necessitated revision surgery due to glioma complications. This contrasts with findings from a recent study of 231 patients with diffuse lower-grade gliomas, where AE rates soared to nearly 50%. However, it is important to clarify that these AEs primarily involved new or worsened neurological deficits [14]. In a different retrospective analysis focusing on malignant gliomas and relying on administrative data, the overall AE rate was substantially lower, recorded at 3.4% [4]. The most frequent surgical complications in this study were iatrogenic-induced strokes (16.3%) and postoperative hematomas (10.3%). In our research, the occurrence of ischemic strokes was not explicitly documented as a separate complication but was noted in cases presenting with clinical symptoms, such as new neurological deficits. Despite this different approach to documentation, we observed that dural leakage and postoperative hematoma were prevalent complications in our cohort, with 2.4% of cases requiring revision surgery for hematoma evacuation. This aligns with findings from Tanaka et al., who reported a 5.6% incidence of postoperative hemorrhage following glioma surgery, including one fatal case, similar to our observations [31]. The relatively high incidence of CSF leakage in our cohort might be partly attributed to the involvement of junior physicians in surgical closures, a stage where inadvertent leakage can occur. This observation underscores the well-known learning curve in neurosurgery [33]. Nevertheless, training emerging surgeons remains a crucial responsibility for academic tertiary care centers. Consequently, some AEs, particularly those associated with the learning process, might be challenging to avoid entirely. To potentially mitigate these training-related AEs, we propose specific measures such as mandatory joint reviews of surgical outcomes by both senior and junior surgeons. Such collaborative evaluations could potentially accelerate the learning curve for individual procedures. However, the effectiveness of these measures in reducing AEs needs to be validated through prospective studies.

Meningiomas, constituting approximately one-third of all primary central nervous system tumors, are the most commonly occurring intracranial neoplasms [28]. Neurosurgical resection remains the cornerstone of meningioma management. However, this surgical intervention is not without risks, notably the potential for new neurological deficits or exacerbation of existing symptoms in patients. The incidence of AEs following meningioma surgery has been reported to range from approximately 10.0 to 25.0%, reflecting significant variability across studies [5, 11, 28, 35]. In a notable study by Schipmann et al., encompassing 500 meningioma patients, the researchers observed a reoperation rate of 9.6%, primarily due to complications such as CSF leakage and surgical site infections [29]. Similarly, Jenkins et al. conducted a prospective analysis on 345 meningioma patients, documenting major AEs in 20.6% of cases. These AEs were characterized by new neurological deficits post-surgery or the necessity for further intervention or reoperation [18]. Specifically, they reported that 1.4% of cases required surgical intervention due to postoperative hematomas, while only 0.3% needed revision surgery for CSF leakage. Our study observed parallel trends, with dural leakages accounting for 0.5% of cases, all necessitating surgical revision. Postoperative hematoma was diagnosed in 1.1% of cases, with 0.8% undergoing surgical revision. Remarkably, our overall surgery-related AE rate was 5.2%, aligning closely with Jenkins et al.’s findings. A critical distinction in our study was the clear separation between surgery-related and non-surgery-related AEs. This differentiation is vital for guiding surgeons in understanding and mitigating potential risks in future operations. Contrarily, amalgamating surgery- and non-surgery-related AEs, as observed in some of the mentioned studies, can lead to ambiguity. This lack of clarity obscures the underlying causes and possible resolutions of these AEs, potentially hindering the development of targeted strategies to reduce their incidence. In summary, our study contributes to the growing body of evidence on the risks associated with meningioma surgery, emphasizing the importance of precise categorization of AEs for enhancing surgical outcomes and patient care.

In our study, non-surgical related adverse events (AEs) were observed in 13.9% of cases, with pneumonia and pulmonary embolism being the most prevalent at 1.6% and 1.7%, respectively. Notably, two patients succumbed to pulmonary embolism. This finding is consistent with previous literature, which reports similar prevalence of non-surgical related AEs. For example, Schipmann et al. identified a 12.1% incidence of nosocomial infections among 2511 individuals undergoing cranial surgery. Their study also highlighted advanced age and a compromised baseline health status as potential predictors for these AEs and subsequent patient readmissions [29]. Additionally, thromboembolic events have been recognized as a frequent cause of patient readmission, with some studies citing a prevalence as high as 19.7% [9, 27]. It is important to underscore that patient factors such as age and baseline health status warrant careful consideration, and in some cases, proactive intensive care unit (ICU) admission may be necessary to enhance patient monitoring. In our cohort, there was a notable requirement for secondary transfers to the ICU or intermediate care (IMC), suggesting the importance of such specialized care in preventing further neurological and systemic deterioration, thereby improving overall medical outcomes. However, routine admission to the ICU should not be standardized as it could lead to resource overutilization. Instead, the development and implementation of ICU scoring systems for judicious patient selection are imperative. This approach aligns with findings from previous studies, which advocate for the careful selection of patients for ICU admission based on specific criteria to optimize care and resource allocation [12, 15, 26]. Therefore, our study not only aligns with previous research in terms of the prevalence of non-surgical related AEs but also emphasizes the need for tailored patient care strategies, particularly in the context of ICU admissions, to ensure optimal patient outcomes.

In this study, the mortality rate was remarkably low, at only 0.8%. This included two instances of death due to pulmonary embolism, two cases where patients succumbed to tumor progression in the absence of viable curative options, and one case of fatal postoperative hemorrhage. Compared to existing literature, where mortality rates in similar settings range from 1.1 to as high as 16.0% [3, 9, 21, 29], our findings represent a significant deviation. We attribute this favorable outcome partly to our rigorous approach in documenting AEs. In our department, cases with complexities undergo thorough review sessions involving the entire neurosurgical team. These meetings focus on pinpointing and addressing any gaps in patient care that could potentially lead to AEs. This proactive strategy is aimed at mitigating the occurrence of future AEs. Additionally, our adoption of the Clavien-Dindo classification system for standardizing AE documentation has not only streamlined our internal processes but also enhanced the comparability of our data with that from other neurosurgical centers [7, 8, 23]. MMCs form a cornerstone of our practice. These conferences serve as platforms for surgical teams to exchange insights, engage in critical analysis, and learn from past errors, thereby preventing recurrence of similar issues [13, 19]. The protocol mandates a detailed examination of AE causes, encompassing both clinical and surgical errors, and extends to evaluating systemic healthcare issues. Furthermore, these discussions are instrumental in the professional development of our residents, as they receive guidance and feedback from seasoned physicians, who scrutinize patient treatment plans to identify areas of strength and improvement [19]. This comprehensive and reflective approach is likely a key contributor to the notably low morbidity and mortality rates observed in our study.

## Limitations

One of the primary strengths of our research represents the effort to analyze AEs using a prospective database encompassing a diverse range of intracranial tumors. Despite its contributions, our study is not without limitations. Our study design precludes the documentation of adverse events occurring beyond the immediate 30-day postoperative window. Additionally, while each case was meticulously overseen, there remains a possibility that certain events may have been misinterpreted. Due to the parameters of our study design, we were unable to determine the discharge destinations of the patients. We acknowledge this as a limitation and suggest that future studies incorporate this variable to more comprehensively evaluate clinical outcomes post-surgery.

Another aspect that could potentially influence our findings is the lack of differentiation between initial surgeries and repeat interventions due to tumor recurrence. This distinction, or lack thereof, might have impacted our results. Moreover, the study did not quantitatively measure the impact of educational interventions during MMCs, leaving the effect of such educational measures on our findings open to interpretation. Our study design precludes the documentation of adverse events occurring beyond the immediate 30-day postoperative window. Although this may influence the observed prevalence of AEs, we contend that the 30-day timeframe is sufficient for a robust evaluation of surgical outcomes. In our investigation, the recorded prevalence of infratentorial tumors was 56%, a figure that notably exceeds traditional epidemiological expectations. This observation suggests the possibility of a selection bias within our dataset, or it may reflect a distinct epidemiological trend within the German cohort under study. Furthermore, to contextualize our findings, we have reviewed the latest studies on the incidence of infratentorial tumors. While direct data for Germany are not readily available in the public domain, studies from other regions may offer some insight. For example, a study conducted in England from 1995 to 2017 reported infratentorial tumors, such as cerebellum and brain stem tumors, accounted for a smaller proportion of primary brain tumors compared to supratentorial sites [34]. In contrast, a study focusing on infratentorial brain tumors noted the complexity of these tumor types and the surgical outcomes associated with them, highlighting the diversity of tumor incidence and behavior across different age groups and populations [1]. Although these studies do not directly reflect the German population, they underscore the variability that can occur in tumor distribution. Given this context, our findings may reflect a unique epidemiological profile within our German cohort or could be indicative of evolving patterns in tumor incidence. The potential for underreporting wound events exists due to the possibility of their management by family practitioners or at other clinics, which our study may not have captured.

## Conclusions

This study represents a significant contribution to the field quality management in neurosurgery, particularly in understanding and documenting AEs in the treatment of intracranial tumors. Our research stands out as a prospectively compiled database, encompassing a wide array of intracranial tumors, to analyze AEs. The findings have highlighted a notably low mortality rate in our cohort, suggesting that meticulous AE documentation and proactive intervention strategies in neurosurgical practice can lead to improved patient outcomes. The insights gained not only contribute to the existing body of knowledge but also pave the way for future research aimed at further improving patient care in the treatment of intracranial pathologies.

## Data Availability

The datasets generated during and/or analyzed during the current study are available from the corresponding author on reasonable request.

## Code availability

The code supporting the findings of this study is available from the corresponding author upon reasonable request.
